# Innovations to Address Depression in High Resource Needs Older Adults

**DOI:** 10.1093/geroni/igaf122.1990

**Published:** 2025-12-31

**Authors:** Jason Burnett, Jennifer Beauchamp, Melba Hernandez-Tejada

## Abstract

This presentation highlights the implementation and preliminary outcomes of multiple studies aimed at improving mental health care access and treatment for older adults. A four-arm randomized controlled trial (RCT) examines behavioral activation (BA) and falls prevention (FP) interventions for homebound older adults with depression, delivered via video conferencing. Recruitment challenges include self-stigma and physical health crises, but qualitative feedback has been positive. Additionally, a feasibility study evaluated the use of Amazon Alexa Show 8 (AES8) devices among cognitively impaired homebound older adults, finding improvements in technology acceptance, depression, and social engagement. These findings informed a subsequent pilot study on remote AES8 installation to combat loneliness and isolation. Another RCT explores tele-delivered BA for post-stroke depression (PSD) prevention in low-income stroke survivors with subthreshold depression. Participants receive five weekly sessions and two booster calls, while the control group receives usual care. The study assesses PSD progression, anxiety, quality of life, and healthcare utilization. Lastly, a study on self-neglect (SN) in older adults—commonly linked to untreated depression—investigates whether intergenerational social connections improve mental health referral acceptance. Preliminary data suggest that pairing APS SN clients with health professional students for weekly calls fosters meaningful connections, leading to increased treatment engagement. Collectively, these studies underscore the role of technology, social engagement, and behavioral interventions in addressing geriatric mental health challenges. Findings support innovative, scalable approaches to enhance access to mental health care, reduce isolation, and improve outcomes for high-needs older adult populations.

